# PIP5 Kinases Regulate Membrane Phosphoinositide and Actin Composition for Targeted Granule Secretion by Cytotoxic Lymphocytes

**DOI:** 10.1016/j.immuni.2018.08.017

**Published:** 2018-09-18

**Authors:** Christian M. Gawden-Bone, Gordon L. Frazer, Arianne C. Richard, Claire Y. Ma, Katharina Strege, Gillian M. Griffiths

**Affiliations:** 1 Cambridge Institute for Medical Research, Cambridge Biomedical Campus, Cambridge CB2 0XY, UK; 2 Cancer Research UK Cambridge, Cambridge Biomedical Campus, Cambridge CB2 0RE, UK

**Keywords:** phosphatidylinositol 4-phosphate 5-kinase, PIP5K, secretion, cytotoxic T lymphocytes, CTLs, immunological synapse, primary cilia, phospholipids, phosphoinositides phosphatidylinositol 4,5-biphosphate, PI(4,5)P2

## Abstract

How cytotoxic T lymphocytes (CTLs) sense T cell receptor (TCR) signaling in order to specialize an area of plasma membrane for granule secretion is not understood. Here, we demonstrate that immune synapse formation led to rapid localized changes in the phosphoinositide composition of the plasma membrane, both reducing phosphoinositide-4-phosphate (PI(4)P), PI(4,5)P2, and PI(3,4,5)P3 and increasing diacylglycerol (DAG) and PI(3,4)P2 within the first 2 min of synapse formation. These changes reduced negative charge across the synapse, triggering the release of electrostatically bound PIP5 kinases that are required to replenish PI(4,5)P2. As PI(4,5)P2 decreased, actin was depleted from the membrane, allowing secretion. Forced localization of PIP5Kβ across the synapse prevented actin depletion, blocking both centrosome docking and secretion. Thus, PIP5Ks act as molecular sensors of TCR activation, controlling actin recruitment across the synapse, ensuring exquisite co-ordination between TCR signaling and CTL secretion.

## Highlights

• Immune synapse formation triggers rapid changes in the membrane composition and charge
• PIP5K is a molecular sensor of TCR activation and is rapidly depleted at the synapse
• PIP5K distribution controls actin recruitment across the immune synapse
• Membrane specialization controls accessibility for centrosome docking and secretion

## Graphical Abstract

**Figure:**
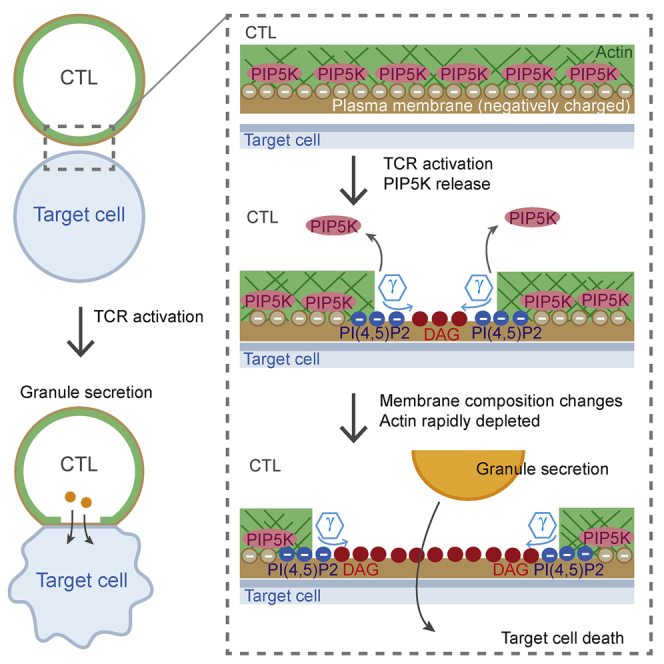

## Teaser

Gawden-Bone et al. show that during immune synapse formation, rapid depletion of PIP5K at the synapse triggers subsequent changes in membrane composition and actin dynamics to establish a zone of localized granule secretion.

## Introduction

Cytotoxic T lymphocytes (CTLs) provide an important line of defense in the immune system, patrolling the body to seek out and destroy virally infected and cancerous cells. They do so with remarkable precision, leaving healthy cells unharmed as they recognize their targets. This is in large part due to a specific mechanism of polarized secretion directed by the centrosome, which docks at the point of contact on the plasma membrane (Stinchcombe et al., 2015), focusing cytotoxic granules for release at the immunological synapse formed between a CTL and its target (Stinchcombe and Griffiths, 2007, Stinchcombe et al., 2006). Secretion is tightly regulated and recent studies have revealed a critical role for the actin cytoskeleton in controlling both the initiation and termination of secretion (Carisey et al., 2018, Hsu et al., 2016, Ritter et al., 2015, Ritter et al., 2017). However, it remains unclear how CTLs translate T cell receptor (TCR) signaling into rapid changes in the membrane, allowing for the tightly orchestrated secretion of granules during TCR activation.

Early imaging studies revealed that the first contacts between CTL and target occur via many small projections (Kalina and Berke, 1976, Sanderson, 1976, Sanderson and Glauert, 1977, Sanderson and Glauert, 1979) that extend from the front of migrating T cells (Ritter et al., 2015). Strikingly, the initial highly interdigitated interface between CTL and target rapidly flattens upon the initiation of TCR signaling to give an extended area of tight membrane contact as the immunological synapse forms (Jenkins et al., 2014). These findings suggest that changes in the composition of the plasma membrane might underlie this transition in membrane structure and raise the possibility that membrane composition might be important for centrosome docking and granule delivery.

Phosphoinositides and other phospholipids play important roles in protein recruitment, signal transduction, and plasma membrane specialization in cells (Di Paolo and De Camilli, 2006, Trimble and Grinstein, 2015). Phosphatidylinositol-4,5-bisphosphate (PI(4,5)P2) is the most abundant phosphoinositide, estimated to comprise ∼2% of the plasma membrane in lymphocytes (Lemmon, 2008, Ross et al., 2016). The relative percentages of different phosphoinositides within the plasma membrane are controlled by a series of kinases and phosphatases (Figure S1A; reviewed in Balla, 2013, Kolay et al., 2016, Ooms et al., 2009, Pirruccello and De Camilli, 2012). Regulating PI(4,5)P2 is particularly important as PI(4,5)P2 binds a variety of actin-regulating proteins and in this way regulates actin dynamics across the plasma membrane (Di Paolo and De Camilli, 2006, Janmey and Lindberg, 2004, Pollard and Borisy, 2003, Raucher et al., 2000, Rohatgi et al., 2000). A number of observations support the idea that there are significant changes in the distribution of phosphoinositides and phospholipids in the plasma membrane as the synapse forms. First, TCR signaling triggers recruitment and activation of phospholipase Cγ1 (PLCγ1), which metabolizes PI(4,5)P2, generating diacylglycerol (DAG) that accumulates on the inner leaflet of the CTL plasma membrane in the synapse, playing an important role in centrosome polarization (Quann et al., 2009, Spitaler et al., 2006). Second, PI(3,4,5)P3 becomes restricted to the periphery of the synapse in T cells (Le Floc’h et al., 2013). Third, in keeping with the role of PI(4,5)P2 in recruitment of F-actin, the depletion of PI(4,5)P2 is accompanied by a loss of F-actin across the synapse prior to centrosome docking and cytolytic granule secretion (Ritter et al., 2015, Ritter et al., 2017).

Centrosome docking at the plasma membrane is unusual in cells but is required for ciliogenesis. Recent reports have shown remarkable similarities between the mechanism of centrosome docking during ciliogenesis and immune synapse formation (Stinchcombe et al., 2015, Tanos et al., 2013). Membrane specialization has been reported in primary cilia, with PI(4,5)P2 and PI(3,4,5)P3 found only at the base of the cilium while PI(4)P is found in the ciliary body (Chávez et al., 2015, Garcia-Gonzalo et al., 2015, Nakatsu, 2015, Phua et al., 2018). This raises the possibility that centrosome docking might be affected by membrane composition.

Our previous studies have revealed that TCR signaling leads to depletion of actin across the synapse, correlating with decreases in PI(4,5)P2 (Ritter et al., 2015). Given the importance of PI(4,5)P2 in both CTL function and ciliogenesis, we decided to examine the phosphoinositide composition as the immunological synapse forms and ask whether membrane specialization is required for centrosome docking and secretion at the immunological synapse. We find that TCR signaling triggers rapid changes in the phosphoinositide composition across the synapse, with cleavage of PI(4,5)P2 to DAG reducing not only actin recruitment but also the negative charge across the synapse. This change in membrane charge prevents phosphatidylinositol 4-phosphate 5-kinases type I (PIP5Ks) from associating with membranes. This in turn prevents replenishment of PI(4,5)P2 at the synapse, maintaining the level of actin depletion required for secretion. Thus, PIP5Ks act as molecular sensors of TCR activation, allowing localized secretion by controlling the membrane composition at the synapse.

## Results

### PLCγ1 and DAG Accumulate while PI(4,5)P2 and Actin Are Depleted within the First 2 Min after CTLs Contact with Targets

To follow changes in phosphoinositide composition as the immunological synapse forms, we used a panel of live-cell imaging probes capable of distinguishing the plasma membrane pool of phosphoinositides within the PI(4,5)P2 metabolic pathway (Figure S1A), combined with 4D spinning-disc microscopy. In each case we co-expressed the F-actin probe, Lifeact-mApple or Lifeact-EGFP, which provides a biomarker for actin depletion during synapse formation (Ritter et al., 2015).

**Figure 1:**
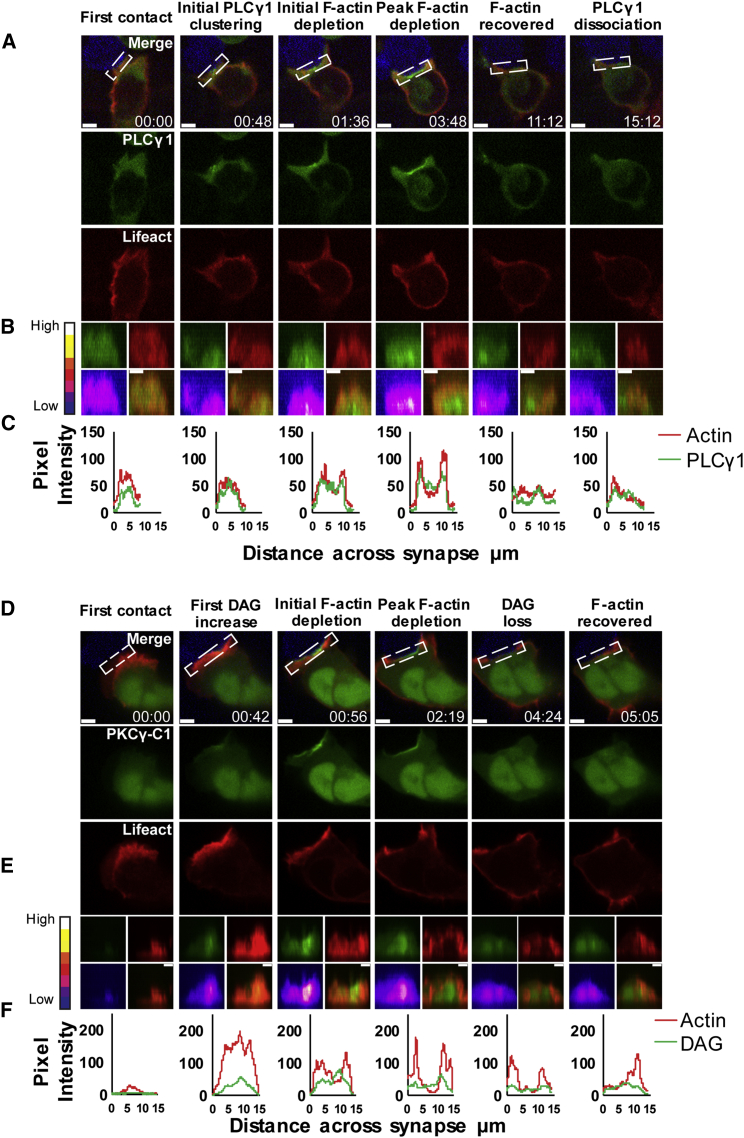
PLCγ1 and DAG Accumulate across the Synapse as It Forms (A–C) CTLs expressing PLCγ1-EGFP and Lifeact-mApple upon first contact with EL4-blue target (t = 0), initial clustering and depletion of probes with time (min:s); 83% conjugates, n = 24; 14 independent experiments. (A) Single confocal slices from Video S1A shown as merged and separate channels. (B) En-face views across 3 μm slice of the synapse as single and merged channels with intensity plot for PLCγ1-EGFP. (C) Pixel intensity (y axis) plots against distance across the synapse (x axis, μm) for boxed region shown in (A). (D–F) CTLs expressing EGFP-PKCγ-C1 to detect DAG and Lifeact-mApple upon first contact between CTL and EL4-blue target (t = 0), initial accumulation and depletion of probes with time (min:s); 100% conjugates, n = 20; 10 independent experiments. (D) Single confocal slices from Video S1B shown as merged and separate channels. (E) En-face views across 3 μm slice of the synapse as single and merged channels with intensity plot for DAG (EGFP-PKCγ-C1). (F) Pixel intensity (y axis) plots against distance across the synapse (x axis, μm) for boxed region shown in (D). Scale bars = 3 μm. See also Figures S1 and S2.

We imaged the initial steps of activation by expressing tagged PLCγ1, observed in the cytoplasm in CTLs (Figure S1B), rapidly translocating to the plasma membrane of the synapse as F-actin (Lifeact-mApple) depleted across the synapse (Figures 1A–1C; Videos S1A and S1B). PLCγ1, which associates with pLAT upon TCR activation (Braiman et al., 2006, Sommers et al., 2002), is recruited to the plasma membrane both in LAT microclusters and independently of LAT (Sherman et al., 2011). We observed the initial accumulation of PLCγ1 at the synapse 48 s after first contact between CTL and target in the example shown, with small fluorescent puncta visible (Figures 1A and 1B). PLCγ1 accumulation expanded across the area of interaction as the initial depletion of actin was detected (1:36) (min:s) (Figure 1C). PLCγ1 intensity increased across the synapse as it formed and were sustained (∼60 units per pixel) until the peak of actin depletion (3:48), diminishing as actin recovered across the synapse (11:12); small puncta of PLCγ1 then re-appeared as CTLs dissociated from targets.

Video S1. Related to Figure 1. PLCγ1 Accumulates at the Synapse during Actin Depletion while DAG Accumulates at the Synapse during Actin Depletion Video S1A corresponding to images in Figure 1A-C with PLCg1-EGP (green); Lifeact (red); targets (blue). Video S1B corresponding to images in Figure 1D-F with EGFP-PKCγ-C1 (DAG; green); Lifeact (red); targets (blue); scale bars = 3 μm.

**Figure 2:**
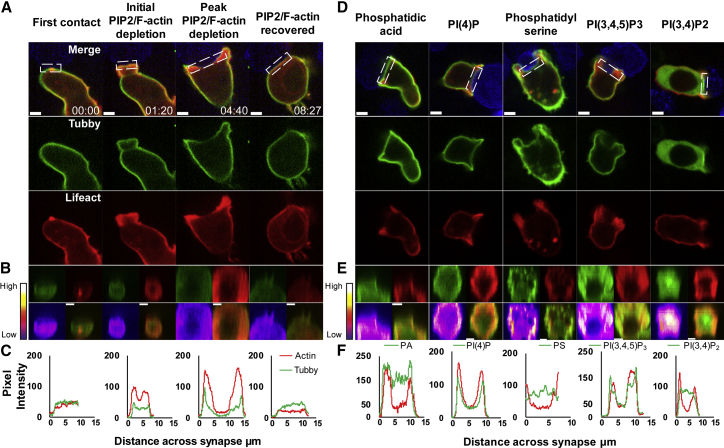
The Immune Synapse Undergoes Membrane Specialization as It Forms (A–C) CTLs expressing EGFP-Tubby and Lifeact-mApple upon first contact with EL4-blue target (t = 0), initial depletion, and recovery of probes with time (min:s); 100% conjugates, n = 27; 14 independent experiments. (A) Single confocal slices from Video S2A shown as merged and separate channels. (B) En-face views across 3 μm slice of the synapse as single and merged channels with intensity plot for PI(4,5,)P2 (Tubby). (C) Pixel intensity (y axis) plots against distance across the synapse (x axis, μm) for boxed region shown in (A). (D–F) CTLs expressing EGFP-tagged probes (as shown) and Lifeact-mApple upon first contact between CTL and EL4-blue target (t = 0), showing initial clustering and depletion of probes with time (min:s) taken from Videos S2B–S2F. (D) Single confocal slices taken from Video S2B (PA, PASS; 100% conjugates n = 9; all independent experiments), Video S2C (PI(4)P, Osh2p-PHx2; 100% conjugates, n = 16; all independent experiments), Video S2D (PS, Lactdherin-C2; 61% conjugates, n = 29; 14 independent experiments), Video S2E (PI(3,4,5)P3, Grp1-PH; 100% conjugates, n = 27; 15 independent experiments), and Video S2F (PI(3,4)P2, Bam32-PH; 70% conjugates, n = 10; all independent experiments) showing probes in green and Lifeact in red; EL4-blue targets in merged channel only. (E) Corresponding en-face views across 3 μm slice of the synapse as single and merged channels with intensity plot for the phosphoinositide probe. (F) En-Face Lifeact and phospholipid probe with pixel intensity plots of probes for boxed region shown in (D). See also Figures S1 and S2.

As PLCγ1 generates diacylglycerol (DAG) by cleavage of PI(4,5)P2, we monitored the production of diacylglycerol (DAG) in the plasma membrane using the EGFP-PKCγ-C1 probe, which detects both the plasma membrane and nuclear pools of DAG. In unstimulated CTLs, only the nuclear pool of DAG was detected (Figure S1C). Upon target cell recognition, DAG accumulates at the synapse at 0:42 after initial contact in the example shown, when actin was enriched across the synapse (Figures 1D–1F; Videos S1A and S1B). DAG accumulation increased as actin depletion began (0:56), distributing across the synapse as actin depleted (2:19) until DAG was no longer detectable by 4:24. The dynamics of PI(4,5)P2 and actin depletion across the synapse followed this same time course, with the depletion of both initially observed 1:20 after initial contact between CTL and target, peaking at 4:40 and recovering simultaneously at 8:27 in the example shown (Figures 2A–2C; Video S2A). Line quantitation of pixel intensity across the synapse demonstrated how the dynamic loss of actin mirrors the loss of PI(4,5)P2 (Figure 2C).

Video S2. Related to Figure 2. PI(4,5)P2, PI(3,4,5)P3, PI(4)P and Actin Are Depleted from the Synapse Simultaneously while PI(3,4)P2 Increases and Phosphatidic Acid and Phosphatidylserine Are Constant Video S2A corresponding to images in Figure 2A-C with EGFP-Tubby (PI(4,5)P2; green). Video S2B corresponding to images in Figure 2D-F with EGFP-PASS (Phosphatidic acid/PtdOH; green). Video S2C corresponding to Figure 2D-F with EGFP-Osh2p-PHx2 (PI(4)P; green. Video S2D corresponding to Figure 2D-F with EGFP-Lactadherin-C2 (Phosphatidylserine; green). Video S2E corresponding to Figure 2D-F with EGFP-Grp1-PH (PI(3,4,5)P3; green). Video S2F corresponding to Figure 2D-F with EGFP-NES-Bam32-PH (PI(3,4)P2, green); in all videos Lifeact (red); targets (blue); scale bars = 3 μm.

By compiling the data from ≥13 videos for each combination of probes shown, we derived mean timings for these events (Table S1). This analysis revealed that PLCγ1 first appeared at the plasma membrane 1:12 ± 11 s after CTL contact with the target, with DAG detected in the same time frame (58 ± 11 s), and the depletion of PI(4,5)P2 apparent at 1:56 ± 36 s together with the loss of F-actin (1:59 ± 22 s). Co-expression of probes for PLCγ1, DAG, PI(4,5)P2, and PI(3,4,5)P3 confirmed the co-ordinated changes in the phosphoinositides across the synapse with DAG accumulating and PI(4,5)P2 and PI(3,4,5)P3 (discussed below) depleting as PLCγ1 clustered at the synapse (Figures S2A–S2D). These results show that dynamic changes in the phosphoinositide composition of the CTL plasma membrane occur within the first 2 min after cell contact as actin is reorganized across the forming synapse.

### The Membrane across the Synapse Undergoes Specialization, with Reduction of PI(4)P, PI(4,5)P2, and PI(3,4,5)P3 and Accumulation of DAG and PI(3,4)P2

The changes in PI(4,5)P2 and DAG seemed likely to affect other plasma membrane phosphoinositides as they lie in the same metabolic pathway (Figure S1A). Therefore, we examined changes in other phosphoinositides before (Figures S1E–S1I) and after synapse formation at the point when actin was depleted (Figures 2D–2F; Videos S2B–S2F). Phosphatidic acid (PA) was evenly distributed across the plasma membrane in CTLs and this distribution did not change upon interaction with target cells, when actin depleted across the synapse. Higher expression at the periphery of the synapse reflected membrane accumulation where CTL actin-rich lamellipodia grasped target cells (see PS images in Figure 2D). Phosphatidylinositol 4-phosphate (PI(4)P) diminished across the synapse as detected using the Osh2p probe (Figures 2D–2F) and was particularly clear when using the P4M probe (Figure S2E; Hammond et al., 2014). In contrast, phosphatidylserine (PS) intensity was maintained at ∼100 units per pixel across the synapse when actin depleted (Figures 2D–2F). Thus, in most instances PA and PS content in the plasma membrane remained unchanged, while PI(4)P decreased together with PI(4,5)P2 as the synapse formed.

We asked whether there was any change in PI(3,4,5)P3 across the synapse in response to PI(4,5)P2 depletion and whether this also affected PI(3,4)P2 and PI(3)P (Figures 2D–2F, S2D, and S2F). Using the Grp1-PH probe, which distinguishes PI(3,4,5)P3 from PI(3,4)P2 (while Akt-PH does not) (Varnai et al., 2017), we confirmed the depletion of PI(3,4,5)P3 across the synapse (Le Floc’h et al., 2013); we were also able to show that this change coincided with depletion of actin across the synapse (Figures 2D–2F). Although the initial binding of the Grp1-PH domain may require interaction with PS in the membrane prior to specific binding to PI(3,4,5)P3 (Lai et al., 2013), our finding that PS remained across the synapse suggests that PS is not playing a role in the differential distribution of Grp1-PH, which thus serves as a true reflection of PI(3,4,5)P3 distribution.

As PI(3,4)P2 is a product of SHIP1 action on PI(3,4,5)P3 (see Figure S1A), we asked whether the depletion of PI(3,4,5)P3 affected PI(3,4)P2 across the synapse using the Bam32 probe (Figure S1A). This revealed that PI(3,4)P2 concentrated at the synapse membrane as actin depleted (Figures 2D–2F), consistent with a role for SHIP1 in the depletion of PI(3,4,5)P3. No changes in PI(3)P were detected at the plasma membrane, with PI(3)P localized on intracellular vesicles both before and after synapse formation (Figure S2F). These vesicles were LAMP1 deficient and did not polarize toward the synapse.

Taken together, these results show that there is a rapid change in membrane composition following the initial hydrolysis of PI(4,5)P2 to DAG by PLCγ1. This results in the formation of an area of membrane that is enriched in DAG and PI(3,4)P2 and depleted in PI(4,5)P2, PI(3,4,5)P3, and PI(4)P, with PA and PS not significantly changed (Figure S2G).

### PIP5 Kinase Distribution Changes as the Immune Synapse Forms

The membrane specialization we observed across the immune synapse bore striking similarities to the phosphoinositide composition in primary cilia which, like the immune synapse, are focal centers for signaling, secretion, and endocytosis, with PI(4,5)P2 and PI(3,4,5)P3 depleted across both. This led us to ask whether similar mechanisms controlled membrane composition during both ciliogenesis and immune synapse formation. The phosphatase Inpp5e is required for membrane specialization during ciliogenesis (Chávez et al., 2015, Garcia-Gonzalo et al., 2015, Nakatsu, 2015, Phua et al., 2018). As the balance of PI(4,5)P2 with PI(4)P can be regulated not only by Inpp5e but also the phosphatase OCRL1 and the three isoforms of PIP5K, α, β, and γ (Figure S1A; Hakim et al., 2012, Kwiatkowska, 2010), we asked whether any of these enzymes might play a role in the regulation of PI(4,5)P2 and actin and thereby control secretion from CTLs.

**Figure 3:**
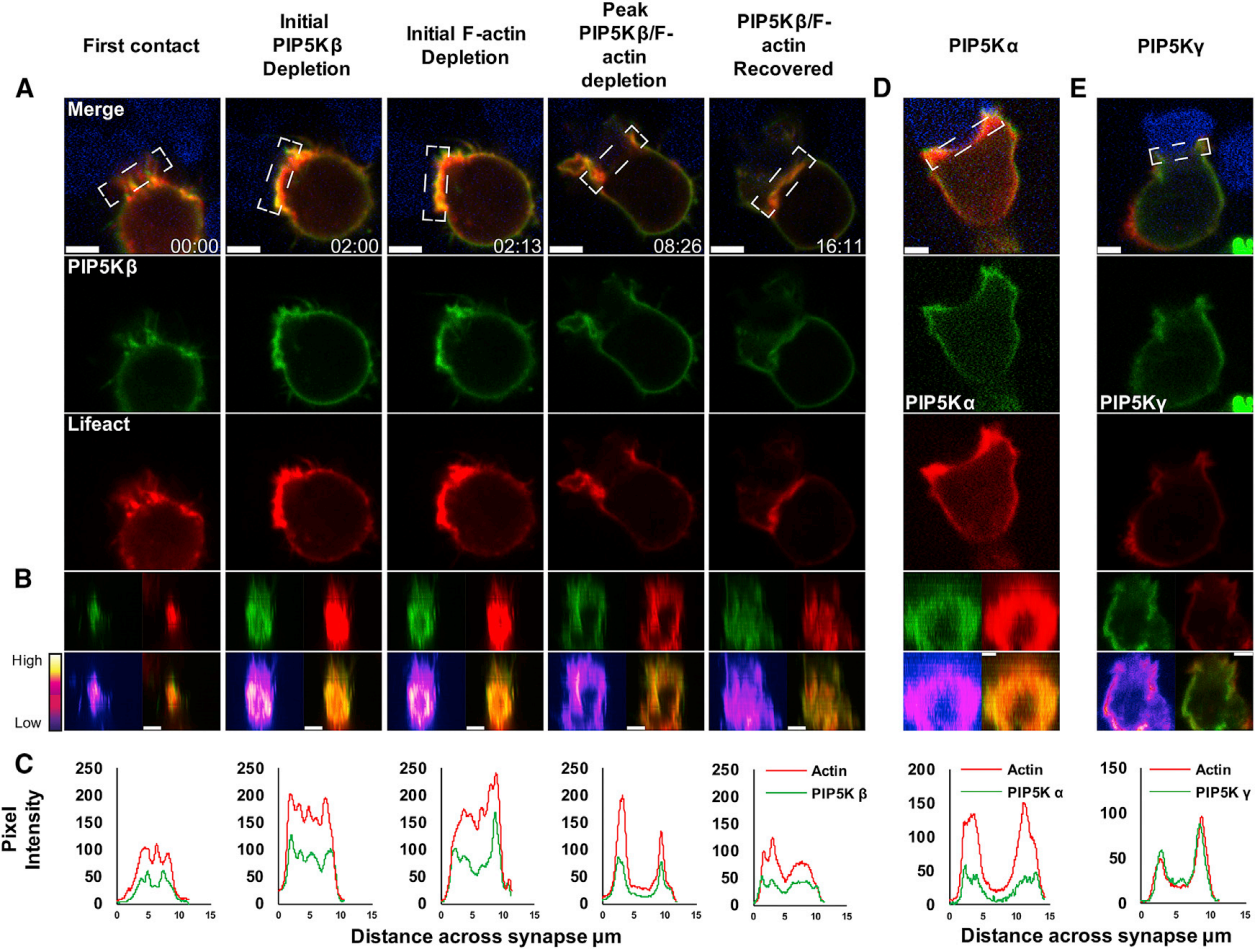
PIP5Kα, PIP5Kβ, and PIP5Kγ Dissociate as Membrane Charge Changes across the Synapse (A) CTL expressing PIP5Kβ-EGFP and Lifeact-mApple upon first contact with EL4-blue target (t = 0), depletion of probes and recovery with time (min:s). Images are single confocal slices from Video S3A shown as merged and separate channels; 100% conjugates, n = 9, all independent experiments. (B) En-face views across 3 μm slice of the synapse as single and merged channels with intensity plot for PIP5Kβ-EGFP. (C) Pixel intensity (y axis) plots against distance across the synapse (x axis, μm). (D and E) CTLs expressing (D) EGFP-PIP5Kα or (E) mCherry-PIP5Kγ (green) and Lifeact-EGFP (red) synapsed with EL4-blue target cells (blue). Images show single confocal slices or en-face images as merged and separate channels from Video S3B (PIP5Kα; 100% conjugates, n = 11, all independent experiments) and Video S3C (PIP5Kγ; 100% conjugates, n = 9, all independent experiments), with an intensity plot for actin and PIP5Ks. Scale bars = 3 μm. See also Figure S3.

Overexpression of the phosphatases Inpp5e, OCRL1 A, or OCRL1 B with Lifeact-mApple revealed a broad cytoplasmic localization and some co-localization with Arf1, consistent with a concentration of both of these phosphatases on the Golgi apparatus (Figure S3). However, there was little overlap with F-actin localization at the plasma membrane. In contrast, all three PIP5K proteins (α, β, and γ, all of which are expressed in CTLs) not only localized to the plasma membrane but also depleted across the synapse together with F-actin, suggesting a potential role in regulating PI(4,5)P2 across the synapse (Figures 3A–3E; Videos S3A–S3C). We mapped the time course of PIP5Kβ using live imaging. In the example shown, the initial depletion of PIP5Kβ at 2:00 slightly preceded actin depletion at 2:13 when viewed en-face (Figure 3B). The pixel intensity plots show that actin depletion peaked at the same time as PIP5Kβ depletion at 8:26 (Figure 3C). Analyzing videos from 22 independent CTL-target cell conjugates gave mean values of 1:39 ± 10 s for the initiation of PIP5K depletion, with actin depleting almost simultaneously at 1:46 ± 11 s and recovering at 8:30 ± 41 s (Table S1).

Video S3. Related to Figure 3. PIP5Kβ, PIP5Kα, and PIP5Kγ Are Depleted as Actin Relaxes at the Synapse Video S3A corresponding to Figure 3A with PIP5Kβ-EGFP (green). Video S3B corresponding to Figure 3A with EGFP-PIP5Kα (green). Video S3C corresponding to Figure 3A with mCherry-PIP5Kγ (green) in all videos CTL express Lifeact (red); targets express Farnesyl-5-TagBFP2 (blue); scale bars = 3 μm.

### PIP5Ks Regulate Actin Recruitment at the Immunological Synapse

**Figure 4:**
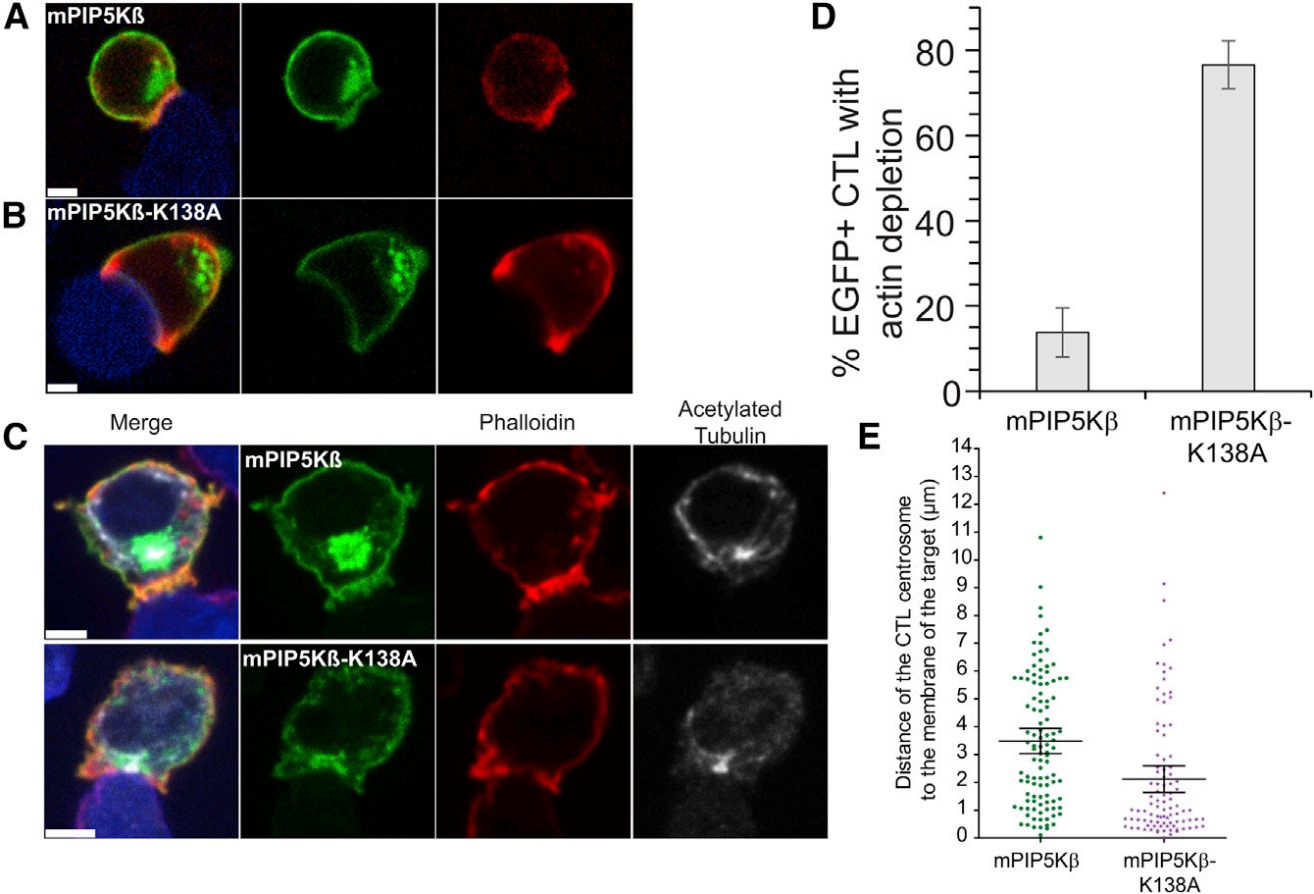
Forced Localization of PIP5K to the Synapse Disrupts Actin Depletion, Centrosome Docking, and Secretion (A) Single confocal slices of CTLs expressing Lifeact-mApple (red) and mPIP5Kβ (green) from Video S4A (representative of all 16 conjugates analyzed, all independent experiments) and (B) kinase-dead mPIP5Kβ-K138A (green) with EL4-blue target cells (blue) from Video S4B. (C) CTLs expressing mPIP5Kβ or mPIP5Kβ-K138A (both green) synapsed with EL4-blue target cells (blue), fixed and stained with Phalloidin-Alexa555 (red) and mouse anti-acetylated tubulin (white), showing single confocal slices from separate channels and merged images. Scale bars = 3 μm. (D) Quantitation of CTLs with actin depletion across the synapse for samples shown in (C). (E) Distance of centrosome to target cell membrane, measured from the center of the acetylated tubulin intensity to the EL4 membrane in CTLs expressing mPIP5Kβ or mPIP5Kβ-K138A (n = 106 and 96, respectively, 3 independent experiments). See also Figure S4.

Given the coincidence between the depletion of PIP5Ks and actin across the synapse, we asked whether PIP5Ks might play a role in the recruitment of actin to the synapse. We used a plasma membrane localization motif that would retain PIP5Ks across the synapse, by tagging PIP5Kβ-EGFP with the N-terminal Lyn-palmitoylation domain (MGCIKSKRKD) (Inoue et al., 2005). This generated a construct, which we named mPIP5Kβ in which the kinase was active, as well as a kinase-inactive form with a point mutation at K138 (mPIP5Kβ-K138A). Expression of these constructs resulted in plasma membrane localization of PIP5K that persisted at the interface between CTLs and targets (Figures 4A and 4B; Videos S4A and S4C). Strikingly, CTLs expressing mPIP5Kβ were often rounded with only 14% (n = 106) showing any F-actin depletion across the synapse (Figure 4C). In contrast, CTLs expressing the kinase-dead mutation showed a typical polarized morphology with 76% (n = 96) showing depleted actin across the synapse (Figure 4C). These results show that altered localization of PIP5K is sufficient to modulate actin depletion across the synapse and this is dependent on its kinase activity.

Video S4. Related to Figure 4. mPIP5Kβ Blocks Actin Depletion across the Synapse while the Inactive Mutant mPIP5Kβ-K138A Does Not Video S4A corresponding to Figure 4A with mPIP5Kβ (green). Video S4B corresponding to Figure 4B with mPIP5Kβ-K138A (green) in both videos cells are also expressing Lifeact (red) and targets are expressing Farnesyl-5-TagBFP2 (blue); scale bars = 3 μm.

### Forced Localization of PIP5K to the Synapse Disrupts Centrosome Docking and Secretion

We next asked whether, by controlling changes in the membrane composition across the synapse, PIP5K also played a role in centrosome docking. Fixed CTL-target conjugates were co-stained with phalloidin to detect F-actin and antibodies against acetylated tubulin to identify the centrosome position. Measurement of the distance from the center of the highest concentration of acetylated tubulin to the synapse revealed that centrosome docking was impaired in CTLs expressing mPIP5Kβ, with only 7% docked (8/106) (<0.5 μm); in contrast, CTLs expressing mPIP5Kβ-K138A in which the kinase is inactive showed efficient centrosome docking with 34% (31/92) docking at the synapse (Figures 4D and 4E). Thus, PIP5Ks have an important role in mediating the membrane changes required for efficient centrosome docking at the immune synapse.

**Figure 5:**
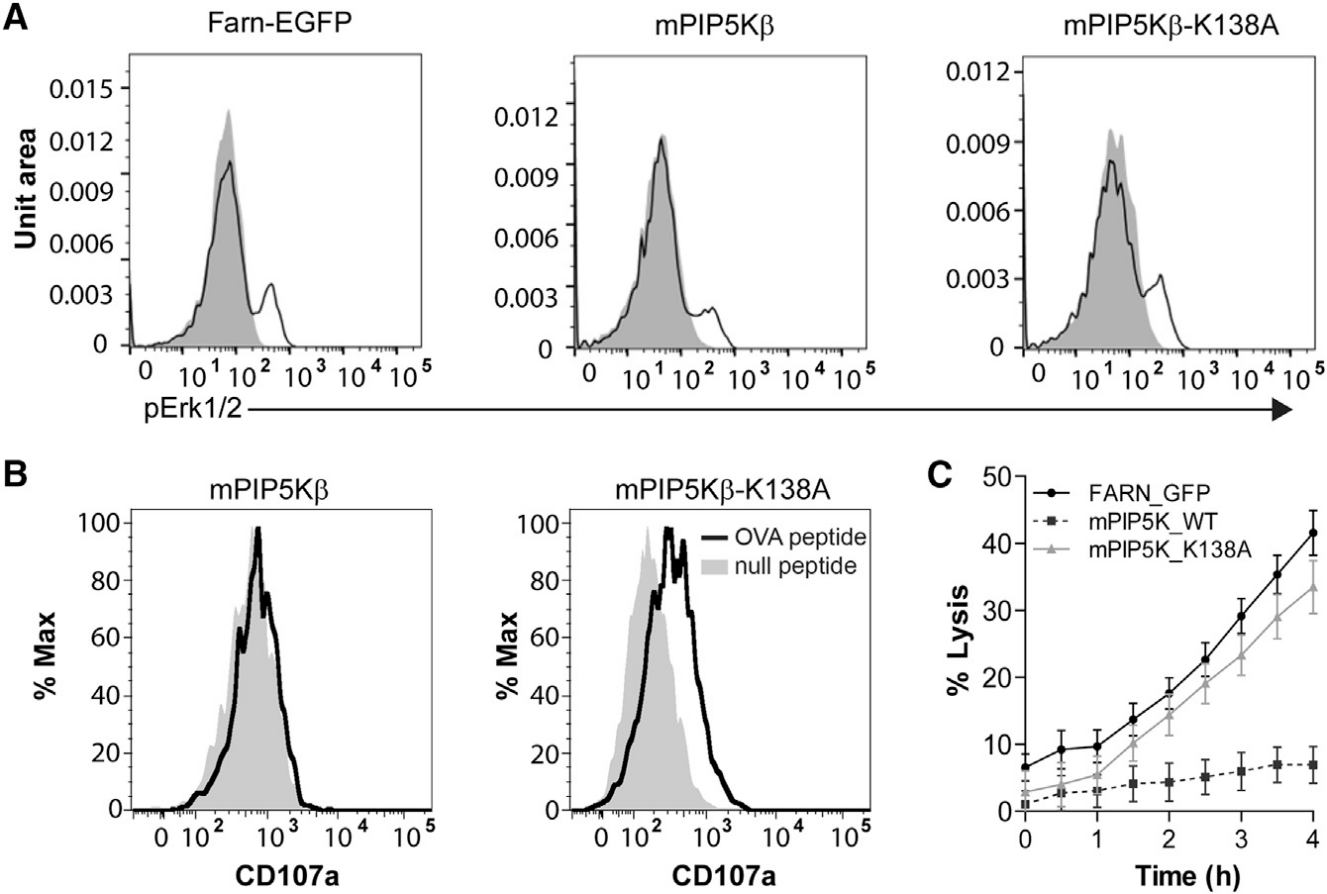
TCR Activation Fails to Induce Degranulation and Target Cell Killing (A) pERK activation of CTLs expressing mPIP5Kβ, mPIP5Kβ-K138A, or farnesyl-EGFP stimulated with plate-bound anti CD3 (black line) or unstimulated (shaded peaks) (n = 3 independent experiments). (B) Degranulation assay measured by CD107a uptake of CTLs expressing either mPIP5Kβ or mPIP5Kβ-K138A in response to targets pulsed with either OVA (bold line) or the null-peptide (NP68) (gray shaded peaks) after 2.5 hr (n = 4 independent experiments). (C) Percentage target cell lysis of NucRed-EL4 over 4 hr by CTL expressing either mPIP5K (dotted-line), mPIP5Kβ-K138A (gray line) or farnesyl-EGFP (black line) (showing propagated SEM of triplicates; n = 3 independent experiments). See also Figure S4.

We next asked whether granule secretion was impaired by forcing PIP5K to remain across the synapse. CTLs expressing mPIP5Kβ and mPIP5Kβ-K138A formed TCR-activated conjugates in which anti-pY demonstrated phosphorylation at the synapse (Figure S4). We were further able to demonstrate that TCR signals were transduced, as activation gave rise to ERK phosphorylation in both mPIP5Kβ and mPIP5Kβ-K138A, as in CTLs expressing control farnesyl-EGFP (Figure 5A). We next asked whether secretion was impaired using a CD107a (Lamp1) degranulation assay (Figure 5B). In CTLs expressing mPIP5Kβ, there was no shift in CD107a signal upon TCR activation with OVA-pulsed targets compared to mPIP5Kβ-K138A-expressing controls, consistent with a defect in degranulation in CTLs expressing mPIP5Kβ (Figure 5B). We confirmed a loss of CTL cytotoxicity using an assay that measured target cell lysis via loss of a red nuclear marker expressed in target cells (NucRed EL4) (Figure 5C). This showed that while CTLs expressing mPIP5Kβ-K138A or the farnesyl-EGFP membrane marker triggered rapid target cell death, CTLs expressing the kinase active mPIP5Kβ failed to kill target cells. These results indicated that forced localization of PIP5K across the synapse disrupts granule secretion.

### PIP5Kα, PIP5Kβ, and PIP5Kγ Are Displaced as Membrane Charge Changes across the Synapse

Given the importance of the redistribution of PIP5Ks across the synapse, we sought to understand the mechanisms controlling membrane localization of PIP5Ks in CTLs. Many different mechanisms have been proposed to regulate PIP5K localization at the plasma membrane based on the ability of PIP5Ks to associate directly with Rac1, ARF6, AP-2, β-arrestin, talin, dishevelled, PA, and PI(4)P (Kwiatkowska, 2010, van den Bout and Divecha, 2009). Studies in macrophages have shown that PIP5K localization can be dictated by membrane charge, with key positively charged amino acids in the activation loop of PIP5K interacting with negatively charged lipid head groups to stabilize PIP5K localization at the membrane (Fairn et al., 2009, Liu et al., 2016).

**Figure 6:**
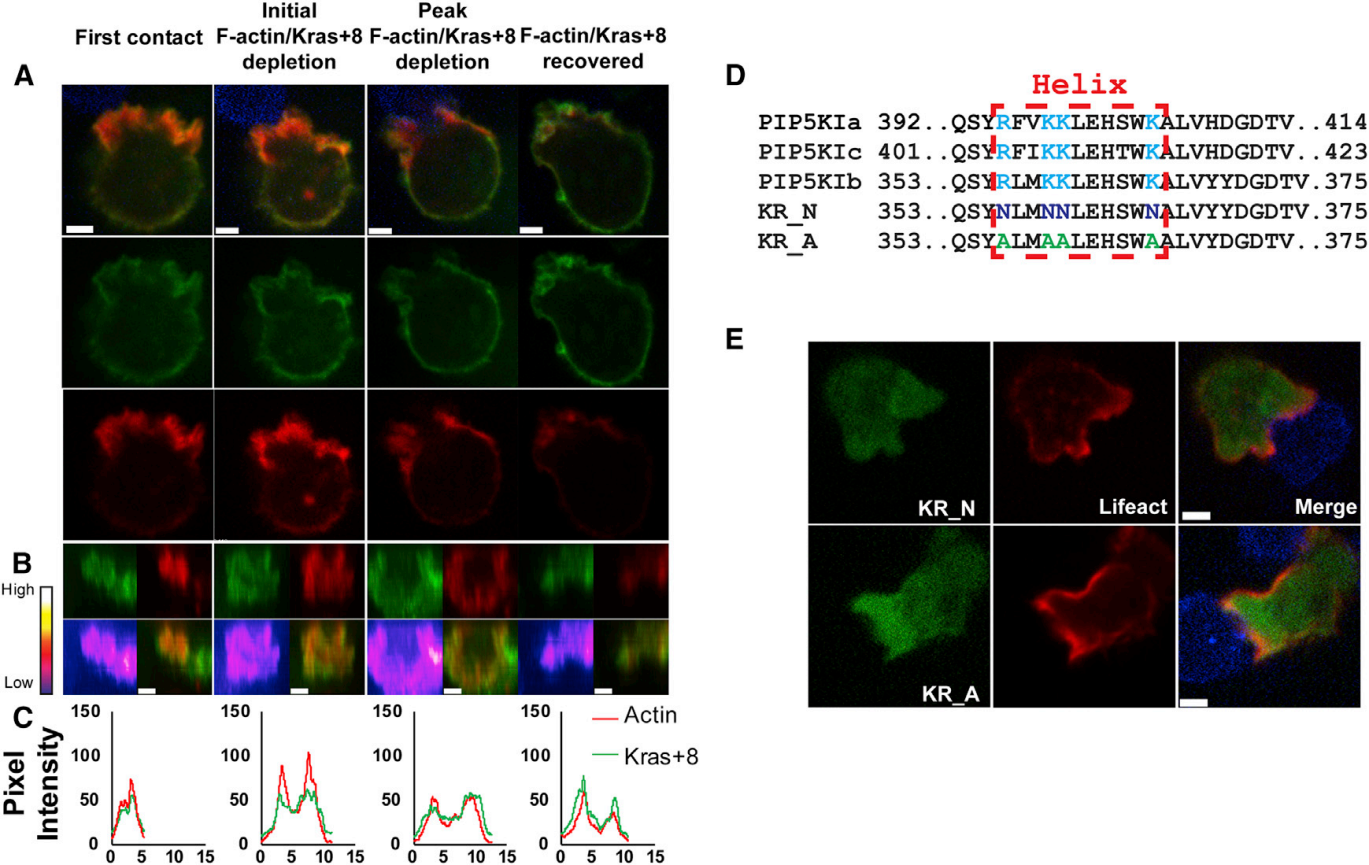
Membrane Charge Regulated PIP5K Distribution in the Synapse (A) CTLs expressing Lifeact-mApple with Kras+8 probe that binds negatively charged areas of the plasma membrane. Upon first contact between CTL and EL4-blue target (t = 0), depletion of probes and recovery with time (min:s). Images are single confocal slices from Video S5A shown as merged and separate channels. (B) En-face views across 3 μm slice of the synapse as single and merged channels with intensity plot for EGFP-Kras+8. (C) Pixel intensity (y axis) plots against distance across the synapse/contact site (x axis, μm). (D) Schematic of the modifications made to the active loop of PIP5Kβ-EGFP. (E) PIP5K β-EGFP KR_N and PIP5Kβ-EGFP KR_A (both green) during depletion of F-actin (red) at the synapse (100% conjugates, n = 10; all independent experiments). Images are single confocal planes taken from Video S5B (PIP5K β KR_N), and Video S5C (PIP5K β KR_A). Scale bars = 3 μm.

As the changes in phosphoinositides that we had observed seemed likely to alter membrane charge (cleavage of PI(4,5)P2 to generate DAG removes three negatively charged phosphate groups), we used a Kras+8 probe that binds to negatively charged membranes (Yeung et al., 2008) to detect changes in charge as the synapse forms (Figures 6A–6C; Videos S5A–S5C). Strikingly, the Kras+8 probe associated with the plasma membrane in CTLs but was depleted in concert with actin as the synapse formed, with the pixel intensity plots for Kras8+ and actin closely matching (Figures 6B and 6C; 100% conjugates, n = 15; 8 independent experiments). Analysis of 15 CTLs provided mean times of 1:56 ± 42 s to the initial loss of binding of the charge probe, with actin depletion observed at 2:14 ± 33 s (Table S1).

Video S5. Related to Figure 6. The Charge Sensor Kras+8 Dissociates from the Membrane during Synapse Formation and Charge Regulates PIP5K Association with the Membrane Video S5A corresponding to Figure 3C with EGFP-Kras+8 (green). Video S5B corresponding to Figure 3D (left) with PIP5Kβ-KR_N-EGFP (green). Video S5C corresponding to Figure 3D (right) with PIP5Kβ-KR_A-EGFP (green) CTL also express Lifeact (red); targets express Farnesyl-5-TagBFP2 (blue); scale bars = 3 μm.

To ask whether electrostatic interactions between PIP5K and the plasma membrane might be responsible for these changes, we mutated the key charged arginine and lysine residues required for electrostatic membrane association of PIP5K to either alanine (A) or asparagine (N) (Fairn et al., 2009, Liu et al., 2016) and examined the localization at the synapse (Figures 6D and 6E; Videos S5A–S5C). Both sets of mutations resulted in a redistribution of PIP5Kβ from the plasma membrane to the cytoplasm in CTLs (Figure 6E). In contrast, a mutation that destroys kinase activity (K138A) (Ishihara et al., 1998) tested in all three PIP5Ks had no effect on membrane localization (data not shown). These results show that electrostatic charge plays a key role in PIP5K association with the plasma membrane in CTLs and reveals a mechanistic basis for the dissociation of PIP5K as electrostatic charge changes across the synapse.

### PIPKs Regulate Membrane Changes across the Synapse

**Figure 7:**
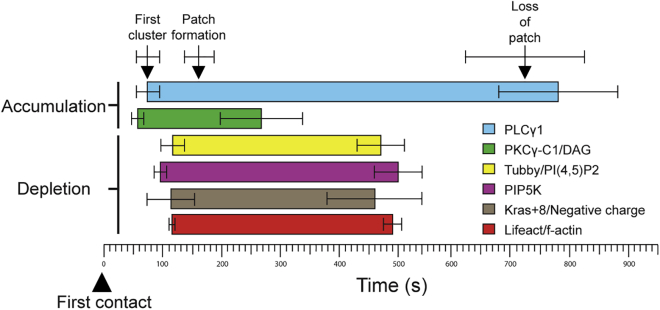
Time Course of PIP5K Association at the Synapse Diagram of the average times (see Table S1) of accumulation (PLCγ1 [blue] and DAG/PKCg-C1 domain [green]) or depletion (Tubby/PI(4,5)P2 [yellow], PIP5K [magenta], Kras+8/negative charge [brown], and Lifeact/F-actin [red]) with the probes subsequent loss or recovery during synapse formation (error bars represent SEM). See also Figure S5.

Compiling our data from multiple videos using each of the probes, we were able to determine mean timings for the dynamic changes in membrane composition across the synapse as it formed (Table S1; Figure 7). This revealed how highly co-ordinated these changes were, with DAG accumulation detected very rapidly after PLCγ1 first clusters at the synapse 1 min after CTL contact with the target. We noted that PLCγ1 often appeared initially as small vesicles forming a cluster before merging into a patch. While the resolution of our imaging for these videos cannot resolve the vesicles further, it is possible that this reflects the co-localization of PLCγ1 with LAT, which is vesicle associated (Sherman et al., 2011). While PLCγ1 remained across the synapse for an extended period, disappearing only after 12 min, the probe does not reveal how long the PLCγ1 remained in its active state.

Our results show that PI(4,5)P2 depletion, the decrease in negative charge, dissociation of PIP5Ks, and loss of F-actin across the synapse occur synchronously, initiating ∼2 min after CTL contact with a target and persisting for ∼2 min (Figure 7; Table S1). These results support a model in which PIP5Ks and F-actin are associated with the plasma membrane. Upon TCR activation, PLCγ1 initiates cleavage of PI(4,5)P2 resulting in the appearance of DAG. The depletion of PI(4,5)P2 is accompanied by a loss of negative charge across this area of membrane, triggering dissociation of PIP5Ks from the membrane, preventing the replenishment of PI(4,5)P2. The PI(4,5)P2-dependent recruitment of actin across this membrane is also lost, allowing both centrosome docking and granule secretion to occur (Figure S5). It is important to note that while PLCγ1 initiates the changes at the synapse, our data clearly show that without the dissociation of PIP5Ks, the membrane changes leading to actin depletion do not occur and neither centrosome docking nor secretion can occur. Our data identify PIP5Ks as sensors of TCR signaling, responding to a TCR-induced change in membrane charge and consequently controlling the membrane composition required for secretion in response to TCR signaling.

## Discussion

How TCR signaling is sensed and rapidly translated into changes that control granule secretion by CTLs is not understood. Although much is known about the changes in receptor and cytoskeletal organization at the synapse, relatively little is known about the corresponding changes in membrane composition and its importance in controlling centrosome docking and secretion. Recent findings have highlighted the role of PI(4,5)P2 in controlling transient actin depletion across the synapse, but the mechanisms controlling these changes have remained elusive. To investigate changes in membrane composition, we used a panel of bioprobes for phospholipids and their modulating lipases, kinases, and phosphatases to track membrane changes as the synapse forms. Within the first minute after CTL target recognition, PLCγ1 is recruited to the synapse, with the appearance of DAG and loss of PI(4,5)P2 and actin occurring within 2 min of contact. While PA and PI(3)P intensities appeared unchanged, PI(4)P, PI(4,5)P2, and PI(3,4,5)P3 decreased and PI(3,4)P2 increased across the synapse. Thus, a highly specialized domain of the plasma membrane is created rapidly as the synapse forms.

These initial changes in membrane composition result in a loss of negative charge across the CTL membrane, triggering dissociation of electrostatically associated PIP5Ks that otherwise would help to maintain this negative charge by generating PI(4,5)P2 from PI(4)P. This loss of PIP5Ks rapidly amplifies the initial PI(4,5)P2 depletion, driving actin dissociation and allowing granule secretion. We show that membrane association of PIP5Ks is charge dependent, as mutation of conserved positively charged amino acids prevented membrane association. However, by forcing continued PI(4,5)P2 generation across the synapse (using a charge-independent localization motif for PIP5Kβ), actin is maintained across the synapse, blocking centrosome docking and granule secretion upon TCR transduction. A reduced negative charge has also been observed in planar views of the Jurkat immune synapse using FLIM and FRET sensors, suggesting that this mechanism may also be used in CD4 T cells (Ma et al., 2017).

We propose a model in which the initial cleavage of PI(4,5)P2 to DAG by TCR-activated PLCγ triggers the loss of membrane charge that is rapidly amplified by the subsequent loss of PIP5Ks (Figure S5). Other mechanisms may also contribute to changes in membrane charge including DAG phosphorylation by diacylglycerol kinases (DGKs) to generate PA. However, DGKα is confined to the periphery of the synapse, thereby serving to limit DAG to the center of the synapse (Chauveau et al., 2014). It is also possible that changes in PS, the most abundant anionic phospholipid in the membrane, might contribute to the loss of negative charge. Quantitation of PS across the synapse proved difficult with PS-rich intracellular organelles closely associated with the plasma membrane such that no clear change could be detected. During phagocytosis, PIP5Ks are displaced in response to a decrease in surface charge limiting local PI(4,5)P2 generation on the forming phagosome (Fairn et al., 2009, Heo et al., 2006, Liu et al., 2016, Yeung et al., 2009). However, PS is maintained on the forming phagosome by PS delivery from endosomes (Yeung et al., 2009). A similar mechanism for regulating PS may also exist at the synapse, and the contribution of PS to the reduced membrane charge across the synapse remains unclear.

Our study highlights the importance of the dynamic PIP5K localization in controlling membrane changes in response to TCR activation. Previous studies have demonstrated the importance of actin dynamics in controlling granule secretion, with actin shown to deplete prior to granule release (Brown et al., 2011, Hsu et al., 2016, Rak et al., 2011, Ritter et al., 2015, Ritter et al., 2017) and to recover rapidly after degranulation (Brown et al., 2011, Hsu et al., 2016, Rak et al., 2011, Ritter et al., 2015, Ritter et al., 2017). Our studies support a role for PIP5Ks in regulating the actin dynamics controlling secretion from CTLs. Actin dynamics may contribute to CTL killing in other ways and it is tempting to speculate that not only the loss of actin but also the changes in phospholipid content of the membrane itself might cause the changes in membrane tension that facilitate CTL killing described in a previous study (Basu et al., 2016).

Specialized domains of phosphoinositides are also observed during phagocytosis and play important roles in recruiting F-actin during formation and sealing of the phagosomal cup, controlled by modulation of PI(4,5)P2. Changes at the immune synapse appear to be a mirror image of those observed during phagocytosis. However, the specialization seen across the synapse is similar to that recently observed in primary cilia, with both PI(4,5)P2 and PI(3,4,5)P3 lost from the main body of the cilium (Chávez et al., 2015, Dyson et al., 2017, Garcia-Gonzalo et al., 2015). As both centrosome docking at the synapse and ciliogenesis are disrupted when membrane composition is perturbed, this raises the possibility that membrane specialization might be required for successful centrosome docking in both contexts.

In conclusion, here we show how TCR signaling rapidly creates an area of plasma membrane specialization with a unique phosphoinositide composition and a reduced negative charge across the immune synapse, causing PIP5Ks to dissociate from the plasma membrane. Thus, PI(4,5)P2 depletion initiated upon TCR activation is perpetuated by the loss of PIP5Ks. The loss of PI(4,5)P2 and the consequent loss of F-actin polymerization facilitates centrosome docking and granule secretion in exact synchrony with TCR signaling, with PIP5Ks acting as sensors of TCR signaling, modifying the plasma membrane to facilitate granule secretion.

## STAR★Methods

### Key Resources Table

**Table:** 

REAGENT or RESOURCE	SOURCE	IDENTIFIER
**Antibodies**		

Mouse monoclonal anti-acetylated tubulin	Sigma-Aldrich	Cat # T7451; RRID: AB_609894
Mouse Anti-Phosphotyrosine Platinum 4G10	Merck/Millipore	Cat# 05-1050X; RRID:AB_916370
Alexa Fluor 647 donkey anti-mouse (H+L)	Thermo Fisher scientific	Cat# A-31571; RRID:AB_162542
Alexa Fluor 405 goat anti-rabbit IgG (H+L)	Thermo Fisher scientific	Cat# A-31556; RRID:AB_221605
Rat anti-CD107a PE	Thermo Fisher scientific	Cat# 12-1071-82; RRID:AB_657556
Rat anti CD16/CD32 (FcR blocking antibody)	Biolegend	Cat# 101301; RRID:AB_312800
Rat anti-CD8 Brilliant Violet 711	Biolegend	Cat# 100747; RRID:AB_11219594
Hamster Anti-mouse CD3ε	BD Bioscience	Cat# 550275; RRID:AB_393572
Rabbit anti-pErk1/2(T202/Y204) D13.14.4E	Cell Signaling Technology	Cat# 4370; RRID:AB_2315112
Rabbit mAb IgG Isotype Control DA1E	Cell Signaling technology	Cat# 3900; RRID:AB_1550038

**Bacterial and Virus Strains**		

NEB 10-beta Competent *E. coli DH10B*	New England BioLabs	C3019

**Chemicals, Peptides, and Recombinant Proteins**		

SIINFEKL (Ovalbumin peptide 257-264)	Anaspec	AS-60193-1
ASNENMDAM (NP68 peptide 366-374)	Anaspec	AS-60623
Recombinant mouse ICAM-1/CD45 Fc Chimera Protein	R&D Systems	796-IC
Alexa Fluor 555-Phalloidin	Invitrogen	A34055
4’,6-Diamidino-2-Phenylindole, Dihydrochloride (DAPI)	Invitrogen	D1306
16% w/v paraformaldehyde	Electron Microscopy Sciences	15710 s
Bovine serum albumin (heat shock fractionation pH 7)	Sigma Aldrich	A7906
Prolong Diamond mounting medium	Invitrogen	P36962
Puromycin	GIBCO/Thermo Fisher	A1113803
Murine Interleukin 2 (IL-2)	Peprotech	212-12
L-Glutamine 200 mM	GIBCO	25030-024
Fetal bovine serum (FBS), heat inactivated	Labtech	FCS-SA
Penicillin/Streptomycin (P/S)	Sigma-Aldrich	P0781
DMEM (+ L-glutamine)	GIBCO	41966029
CO_2_ independent medium (-L-Glutamine)	GIBCO	18045-054
BamHI	Fermentas/Thermo Fisher	FD0054
BshTI	Fermentas/Thermo Fisher	FD1464
Eco91I	Fermentas/Thermo Fisher	FD0394
HindIII	Fermentas/Thermo Fisher	FD0504
KpnI	Fermentas/Thermo Fisher	FD0524
NheI	Fermentas/Thermo Fisher	FD0973
SalI	Fermentas/Thermo Fisher	FD0644
XhoI	Fermentas/Thermo Fisher	FD0694
BsrGI	New England Biolabs	R0575
KpnI	New England Biolabs	R0142

**Experimental Models: Cell Lines**		

EL4-Farnesyl-5-TagBFP2 (EL4-blue)	(Ritter et al., 2015)	N/A
EL4-NucLight-Red	This paper	N/A
EL4-MEM-iRFP670 (FR-EL4)	This paper	N/A
EL4 ATCC TIB39	ATCC	ATCC Cat# TIB-39; RRID:CVCL_0255

**Experimental Models: Organisms/Strains**		

B6.SVJ129-RAG1TM1BAL TG(TCRATCRB)1100MJB	JAX	Cat# JAX:003831, RRID:IMSR_JAX:003831

**Recombinant DNA**		

EGFP-Lactadherin-C2	S. Grinstein	(Yeung et al., 2008)
EGFP-Kras+8	S. Grinstein	(Yeung et al., 2008)
EGFP-Grp1-PH	C. Watts	(Kimber et al., 2002)
EGFP-FYVEx2	H. Stenmark	(Gillooly et al., 2000)
EGFP-OCRL1A/B	M.Lowe	(Choudhury et al., 2005)
mApple-Lifeact-7	Addgene	54747; RRID:SCR_002037
mEGFP-Lifeact-7	Addgene	54610; RRID:SCR_002037
mCherry-PIP5Kγ^661^	Addgene	29584; RRID:SCR_002037
EGFP-P4M-SidMx2	Addgene	51472; RRID:SCR_002037
EGFP-Osh2p-PHx2	T.Balla	(Szentpetery et al., 2009)
EGFP-Tubby	T.Balla	(Szentpetery et al., 2009)
mTagRFP-N	Evrogen	FP142
EGFP-C1, N1 and mCherry-C1	Takara Bio/Clontech	6084-1, 6085-1 & 632524
mPIP5K1β-EGFP	This paper	GeneBank: P25911/P70181
mPIP5K-K138A-EGFP	This paper	GeneBank: P25911/P70181
EGFP-NES-PASS	This Paper	GeneBank: P63248/Q04359
EGFP-NES-Bam32-PH	This paper	GeneBank: P63248/Q9QXT1
EGFP-PKCγ-C1 domain	This paper	GeneBank: P63318
EGFP-PIP5Kα	This paper	GeneBank: P70182
PIP5Kβ-EGFP	This paper	GeneBank: P70181
PIP5Kβ-KR_A-EGFP	This paper	GeneBank: P25911/P70181
PIP5Kβ-KR_N-EGFP	This paper	GeneBank: P25911/P70181
EGFP-Inpp5e	This paper	GeneBank: Q9JII1
PLCγ1-EGFP	This paper	GeneBank: Q62077
PKAi-NES-PASS gBlock	This paper	Integrated DNA Technologies
PKAi-NES-Bam32-PH gBlock	This paper	Integrated DNA Technologies
PKCγ-C1 domain gBlock	This paper	Integrated DNA Technologies
PIP5Kβ K_RA gBlock	This paper	Integrated DNA Technologies
PIP5KβK_RN gBlock	This paper	Integrated DNA Technologies

**Software and Algorithms**		

Imaris	Bitplane	RRID:SCR_007370
Prism 5	Graphpad	RRID:SCR_002798
IncuCyte Spheroid Software Module	Essen BioScience, Sartorius	9600-0019
FlowJo software v10 and v9	Flow Jo LLC treestar	RRID:SCR_008520
ImageJ	ImageJ.net	RRID:SCR_003070

### Contact for Reagent and Resource Sharing

Further information and requests for resources and reagents should be directed to and will be fulfilled by the Lead Contact, Professor Gillian M Griffiths (gg305@cam.ac.uk).

### Experimental Model and Subject Details

C57BL/6 OT-I^Rag1−/−^ mice (RRID:IMSR_JAX:003831) This research has been regulated under the Animals (Scientific Procedures) Act 1986 Amendment Regulations 2012 following ethical review by the University of Cambridge Animal Welfare and Ethical Review Body (AWERB).

### Method Details

#### Tissue culture and cell preparation

Splenocytes from C57BL/6 OT-I^Rag1−/−^ mice (RRID:IMSR_JAX:003831) were isolated by manual rupture of the spleen. Splenocytes were resuspended (∼10^8^ cells/mL) in culture media (RPMI, 10% FCS, 50 U/mL penicillin-streptomycin, 1 mM sodium pyruvate, 2mM L-glutamine, 50μM β2-mercaptoethanol, 100 U/mL IL-2) and incubated with 10 nM SIINFEKL peptide for 3 days at 37°C, 8% CO_2_, washed and cultured for a total of 5-10 days. EL4-MEM-iRFP670 were generated by transduction of EL4 with pMig-MEM-RFP670,. EL4-Farnesyl-5-TagBFP2 (EL4-blue) and EL4-MEM-iRFP670 (FR-EL4) target cells were maintained in DMEM, 2mM L-glutamine, 10% FBS (GIBCO), 50 U/mL pen/strep (EL4 media).

#### RNA preparation, cDNA generation cloning and plasmids

cDNA was synthesized using oligo dT primers from RNA isolated from 5x10^6^ OT-I CTL (3-5 days post-activation) with the RNA mini (QIAGEN) and AffinityScript cDNA synthesis kits (Agilent technologies). PCR amplifications were carried out using AccuPrime Pfx DNA polymerase kits in a G-storm thermal cycler system 482 (G-storm Ltd, Labtech International). Site-directed mutagenesis used the QuickChange II XL Site-Directed Mutagenesis Kit (Agilent Technologies); ligations, the Quick ligation kit (New England Biolabs). Synthesized double stranded DNA were designed then manufactured by Integrated DNA Technologies (gBlocks; IDT, Iowa, USA) and cloned into pSC-B carrier plasmids using the StrataClone Blunt PCR Cloning Kit (Agilent Technologies).

#### Lipid probes

EGFP-Tubby was used to detect PI(4,5)P2. The C1-domains of PKCγ equivalent to amino acids (aa) 35-150 were made into a gBlock and cloned into EGFP-C1 (Clontech) at KpnI and BamHI sites to detect DAG. Likewise, a gBlock corresponding to the phosphatidic acid biosensor with superior sensitivity (PASS), derived from the yeast protein Spo20p phosphatidic acid binding domain (PABD) (Zhang et al., 2014) was also cloned into EGFP-C1 at restriction sites XhoI and SalI. Detection of PI(4)P was with Osh2p (Roy and Levine, 2004). PS was detected with the C2 domain of Lactadherin (Yeung et al., 2008). Detection of PI(3,4,5)P3 used the specific Grp1-PH probe (Kavran et al., 1998). As both Bam32-PH and the TAPP1-PH domains available to detect PI(3,4)P2 showed strong nuclear localization (Anderson et al., 2000, Kimber et al., 2002), we included the nuclear export signal (NES) of Protein Kinase A inhibitor aa 38-47 (LALKLAGLDI) before the Bam32-PH domain aa 164-256. NES-Bam32 was made into a gBlock and inserted into EGFP-C1 at restriction sites BsrGI and KpnI. This improved signal for the plasma membrane pool of PI(3,4)P2. PI(3)P was detected using EGFP-FYVEx2 (Gillooly et al., 2000).

#### Cell preparation for live cell microscopy

1 × 10^7^ OT-I CTLs (5–8 days after activation) were nucleofected 24 h prior to imaging with 5 μg Lifeact-mApple and probe construct plasmids using the Mouse T Cell Nucleofector Kit (Lonza). EL4-blue target cells were pulsed with 1 μM SIINFEKL for 30 min at 37°C, washed in serum-free CO_2_-independent medium (with L-glutamine) and plated onto 35-mm glass-bottom culture dishes (MatTek) coated with 0.5 μg/mL murine ICAM-1/Fc. ∼2 × 10^6^ nucleofected CTLs in CO_2_-independent T cell medium were added dropwise, imaging was started within 5 min. Interactions were imaged with cells in an environmental chamber maintained at 37°C (Okolabs) on an Olympus IX81 microscope (Olympus) using the Andor Revolution spinning-disc microscope with Yokogawa CSU-X1 spinning disk, iXon Ultra 888 EMCCD camera, 2x camera adaptor (Andor Technology, Oxford instruments) and Olympus Universal Plan Super Apochromat silicone immersion objective. 12-18 z stacks (0.8-μm apart) were imaged every 12 s with fluorophores excited at 405, 488, and 561 nm in each z-plane. 4D datasets were rendered and analyzed with Imaris software (Bitplane).

#### Fixed cell preparation, image collection and quantitation

OT-I CTLs (2-6 days post-activation) were nucleofected with 5 μg DNA encoding wild-type mPIP5Kβ or mPIP5Kβ-K138A, mixed with peptide pulsed targets (as above) in serum-free, CO2-independent T cell media at 37°C for 5 min, plated onto glass coverslips (n = 1.5; VWR) and allowed to form conjugates for 20 min. Cells were fixed with 4% v/v paraformaldehyde at 37°C and washed at room temperature with PBS and quenched in Tris buffered saline (TBS) before permeabilization in TBS, 0.2% Triton X-100 (Sigma Aldrich) for 5 min. Samples were blocked with TBS, 2% BSA for 20 min before incubation with mouse anti-acetyl tubulin for 1h. Samples were washed in tris buffered saline and incubated with goat anti-mouse IgG-Alexa 647 and Phalloidin-Alexa fluor 555 for 30 min. Coverslips were washed, mounted with Prolong Diamond mounting medium and imaged on an Andor Revolution spinning-disc microscope (as above) with z stacks at 0.15 μm intervals.

#### Intracellular Phospho-protein Flow Cytometry

Nucleofected cells were sorted for GFP expression, and stimulated with 2 μg/mL plate-bound anti-CD3ε antibody for 60 min at 37°C. Cells were fixed in 4% paraformaldehyde at room temperature for 15 min and washed in PBS. Pre-chilled cells were permeabilized with 90% ice-cold methanol for 30 min on ice. Cells were washed in PBS and resuspended in buffer (1% FBS in PBS) with 1 μg/mL FcR blocking antibody with either 2.5 μg/mL rabbit anti-pERK1/2 antibody or 12.5 μg/mL rabbit IgG isotype control antibody and incubated for 1h at room temperature. After washing in buffer, cells were resuspended in buffer containing 1 μg/mL FcR antibody and 10 μg/mL Alexa Fluor 405 goat-anti-rabbit incubated for 30 min at room temperature. Cells were washed in incubation buffer prior to data acquisition on a BD LSRFortessa (BD Biosciences). The data was analyzed with FlowJo (FlowJo, LLC).

#### Degranulation assay

FR-EL4-were pulsed with 1 μM ovalbumin peptide (SIINFEKL) or control peptide NP68 (ASNENMDAM) for 1 hour at 37°C before washing to remove free peptide. 1x10^5^ CTLs were cultured 1:1 with pulsed FR-EL4 cells for 2.5 h in culture media supplemented with 2 μg/mL anti-CD107a PE in round-bottom 96-well plates. Cells were stained on ice with FCR antibody, DAPI, and anti-CD8 Brilliant Violet 711. Flow cytometry was performed on BD LSRFortessa (BD Biosciences) and data were analyzed in FlowJo.

#### Cytotoxicity assay

EL4 cells expressing a non-perturbing red nuclear marker (NucLight-Red; Essen Bioscience, Sartorius) (NucRed EL4) were pulsed with 1 mM SIINFEKL peptide, washed and incubated with OT-I CTL expressing either mPIP5Kβ-EGFP or mPIP5Kβ-K138A-EGFP or farnesyl-EGFP. Triplicate samples of 20000 CTL were plated with 2000 EL4 cells per well. The assay was performed in an IncuCyte S3 live cell analysis system (Essen Bioscience, Sartorius) maintained at 37°C and 10% CO_2_. Cells were allowed to settle in the plates for 30 min and images were captured every 30 min over 4 h using the 565-605nm laser line and the 4x objective. Loss of NucRed EL4 intensity was quantitated using IncuCyte S3 software with the spheroid quantitation application. Data were exported and calculated as percentage of target cell loss based on the initial target cell number for each well analyzed then plotted using Prism software (Graphpad).

### Quantification and Statistical Analysis

#### Cell choice

Nucleofected OT-I cells were added dropwise to dishes and allowed to settle for less than 5 min. After this point a systematic scan across the dish was undertaken until a cell that was double labeled (expressing both nucleofected plasmids) to detectable level and in close location to the target EL4 blue was selected for filming. In fixed samples a systematic scan was made from the top left of the coverslip to the right and images were taken at either 60 or 100x of the EGFP positive cells for quantitation measurements. Images were analyzed using Imaris software (Bitplane) line tool to determine centrosome-to-synapse distances and > 5-fold depletion of the F-actin signal across the synapse relative to membrane outside the synapse.

#### Quantitation in ImageJ/Fiji

Movies of CTL expressing lipid probes and Lifeact during synapse formation were analyzed using Imaris software. Single representative Z-planes within the projected images through the center of the synapse were captured as Tiff files, opened in ImageJ/Fiji (ImageJ open source software http://Imagej.net) and split into their respective red, green and blue channels. Pixel intensity values were determined across 2.1 μm depth as indicated in each figure to capture the undulating interface across each synapse. “Accumulation” is defined as pixel intensity > pixel intensity at initial contact (t = 0); “peak” intensity as the highest pixel intensity value compared to t = 0 and “recovered” when pixel intensity values return to t = 0. Fold changes vary for different probes as shown in pixel intensity plots for each figure.

#### Error

All error in graphs is standard error of the mean or propagated standard error of the mean where it is stated (Figure 5C)

### Data and Software Availability

Due to the size of the raw dataset it will be made available on request.

## Supplemental Information

Document S1. Figures S1–S5 and Table S1

Document S2. Article plus Supplemental Information
