# Supplementary material for: PIP5 Kinases Regulate Membrane Phosphoinositide and Actin Composition for Targeted Granule Secretion by Cytotoxic Lymphocytes
Source: Immunity. 2018 Sep 18;49(3):427–437.e4. doi: 10.1016/j.immuni.2018.08.017 (PMC6162341; doi:10.1016/j.immuni.2018.08.017)
Supplement: Document S1. Figures S1–S5 and Table S1 [file mmc1.pdf]

Immunity, Volume *49*

Supplemental Information

PIP5 Kinases Regulate Membrane Phosphoinositide and Actin Composition for Targeted Granule Secretion by Cytotoxic Lymphocytes

Christian M. Gawden-Bone, Gordon L. Frazer, Arianne C. Richard, Claire Y. Ma, Katharina Strege, and Gillian M. Griffiths

A

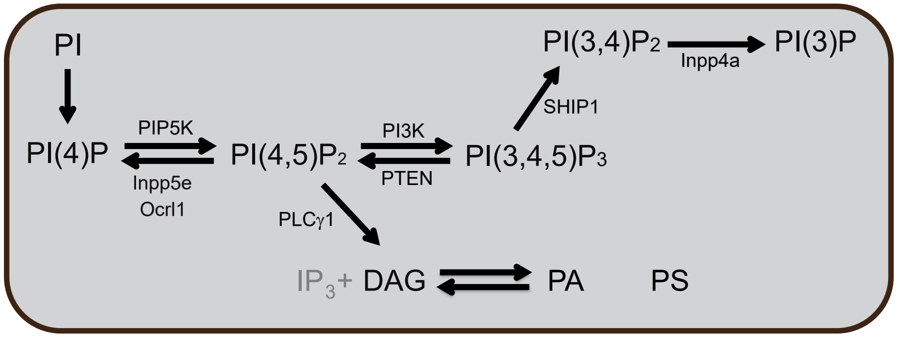

Lipid	Probe
PI(4)P	EGFP-Osh2p-PHx2
PI(4,5)P2	EGFP-Tubby
DAG	EGFP-PKCγ-C1
Phosphatidic acid (PA)	EGFP-PASS
PI(3,4,5)P3	EGFP-Grp1-PH
PI(3,4)P2	EGFP-NES-Bam32-PH
PI(3)P	EGFP-FYVEx2
Phosphatidylserine (PS)	EGFP-Lactadherin-C2

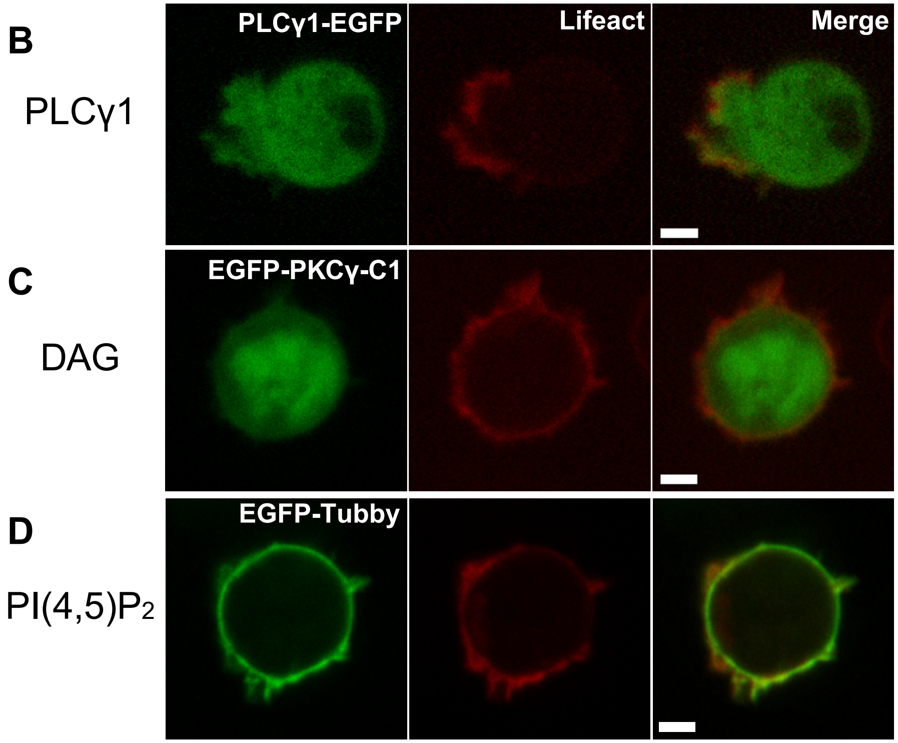

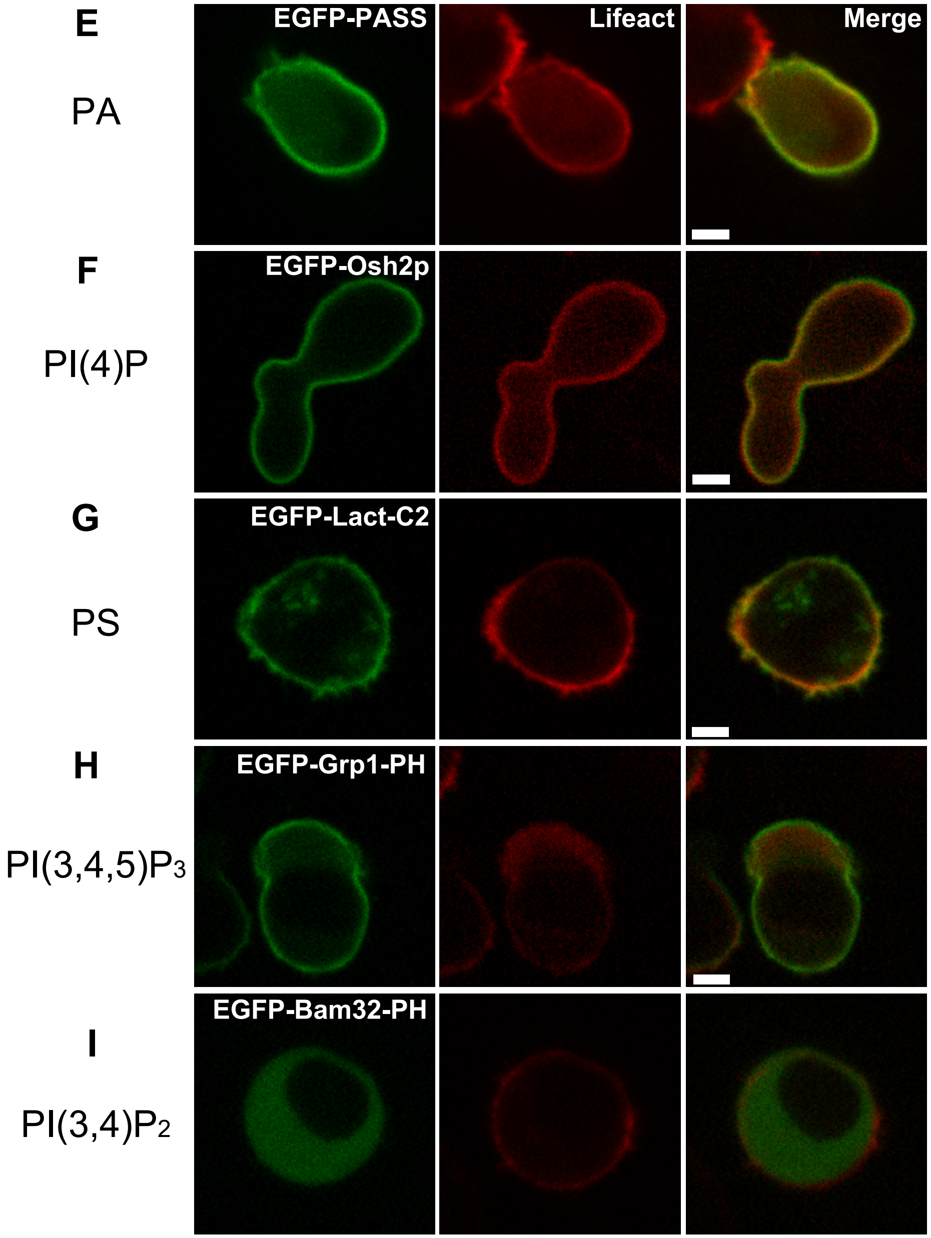

Figure S1. Related to Figure 1 and Figure 2. Bioprobes used to follow phosphoinositide metabolism in the plasma membrane. (A) Schematic of the metabolic pathways regulating plasma membrane phospholipids and the bioprobes used for detection and table of probes used in this study. (B-I) Distribution of phosphoinositides in CTL without targets. Scale bars = 3 µm.

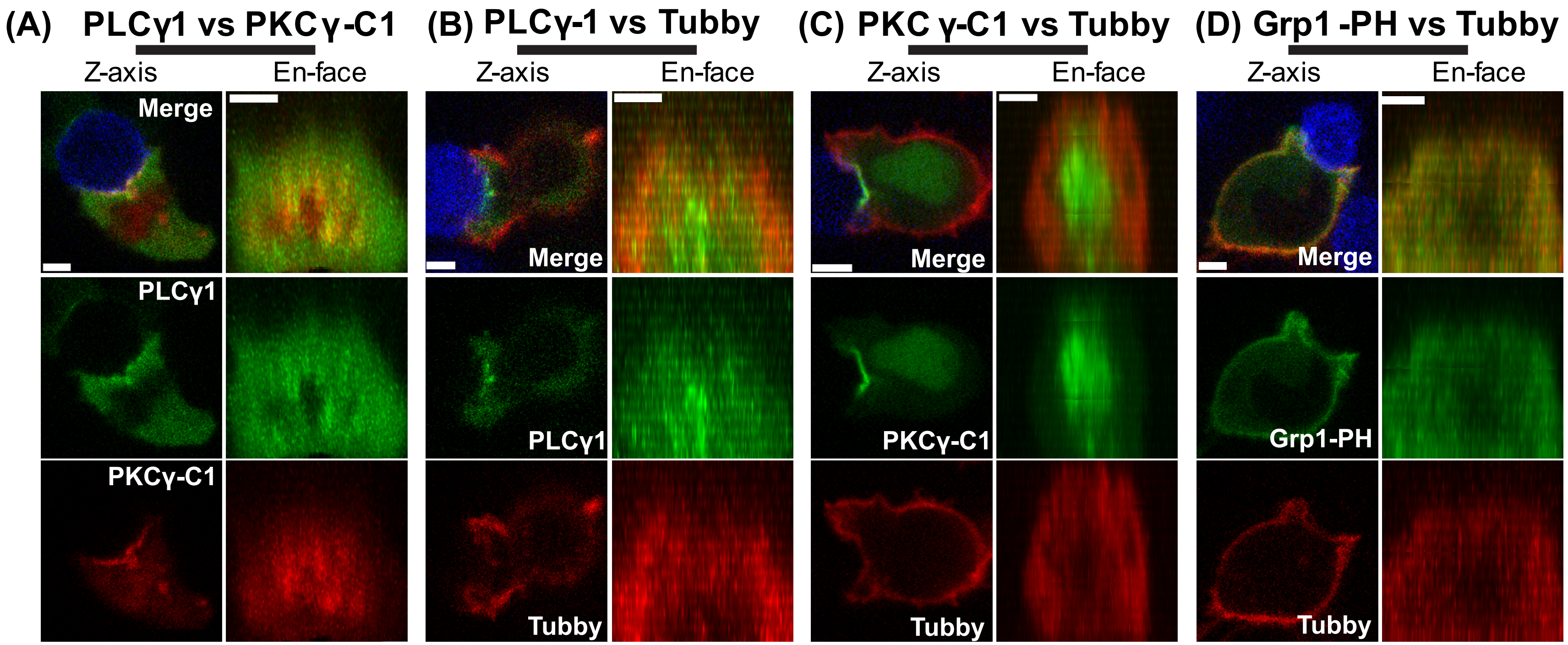

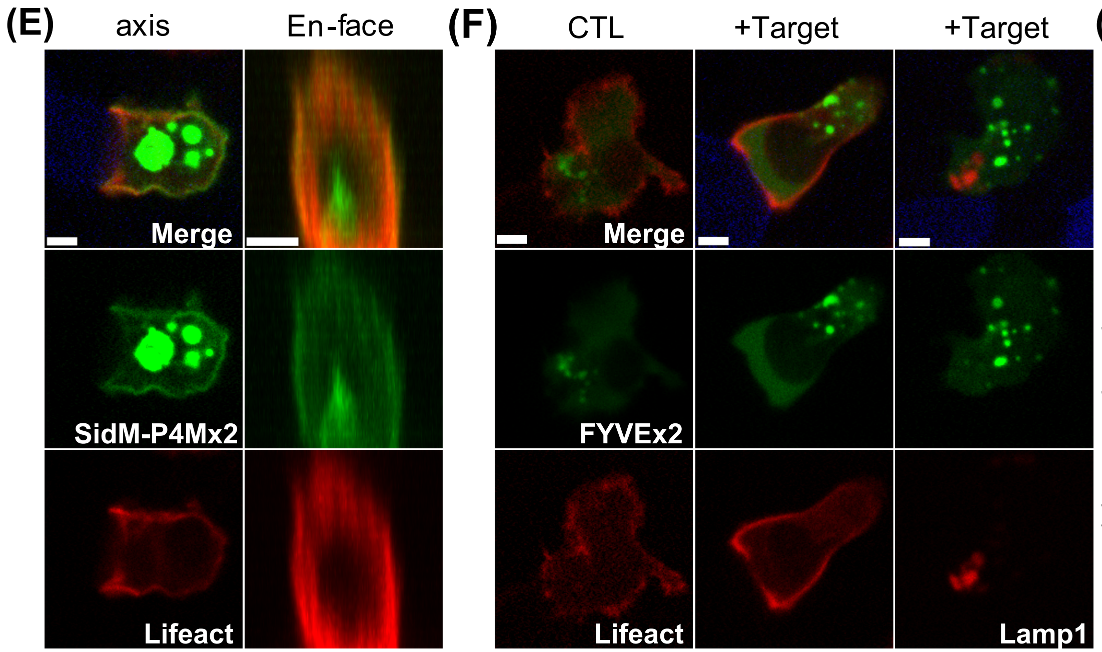

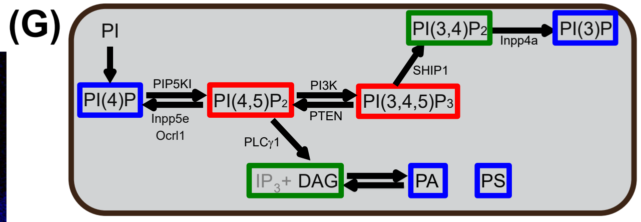

Figure S2. Related to Figure 1 and Figure 2 coincidence of phosphoinositide changes. A) Co-expression of PLCγ1-EGFP (green) and mCherry-PKCγ-C1 domain (red) in CTL with target EL-4 (blue) as single confocal slices and en-face images. This shows colocalisation of PLCγ1 and DAG at the interface. (B) PLCγ1-EGFP (green) and mCherry-Tubby (red; as above) showing PLCγ1 accummunlation and Tubby exclusion at the centre of the synapse. (C) EGFP-PKCγ-C1 domain (green) and mCherry-Tubby (red) (D) EGFP-Grp1-PH (green) and mCherry-Tubby (red). (E) EGFP-P4M-SidMx2 (green) and Lifeact (red), all with target EL4-blue (in merge; z-axis) and en-face sections 3 μm depth across the synapse showing clearly the PI(4)P depletion at the synapse (100% of conjugates, n=8 all independent experiments). In en-face sections (A-E) EL-4 channel is excluded for clarity. (F) Expression of EGFP-FYVEx2 (green) and Lifeact (red) showing PI(3)P is localised intracellularly both in resting CTL (left column; CTL) and in CTL in contact with EL-4 targets (blue; middle column; + Target; 100% conjugates, n=21; 13 independent experiments). The FYVEx2 positive organelles do not colocalize with Lamp1 (red; right column; + Target). (G) Cartoon of the metabolic pathways for phosphoinositides in the plasma membrane summarising the changes that occur upon synapse formation with reduced levels boxed in red, increased in green and unchanged in blue. Scale bars = 3μm.

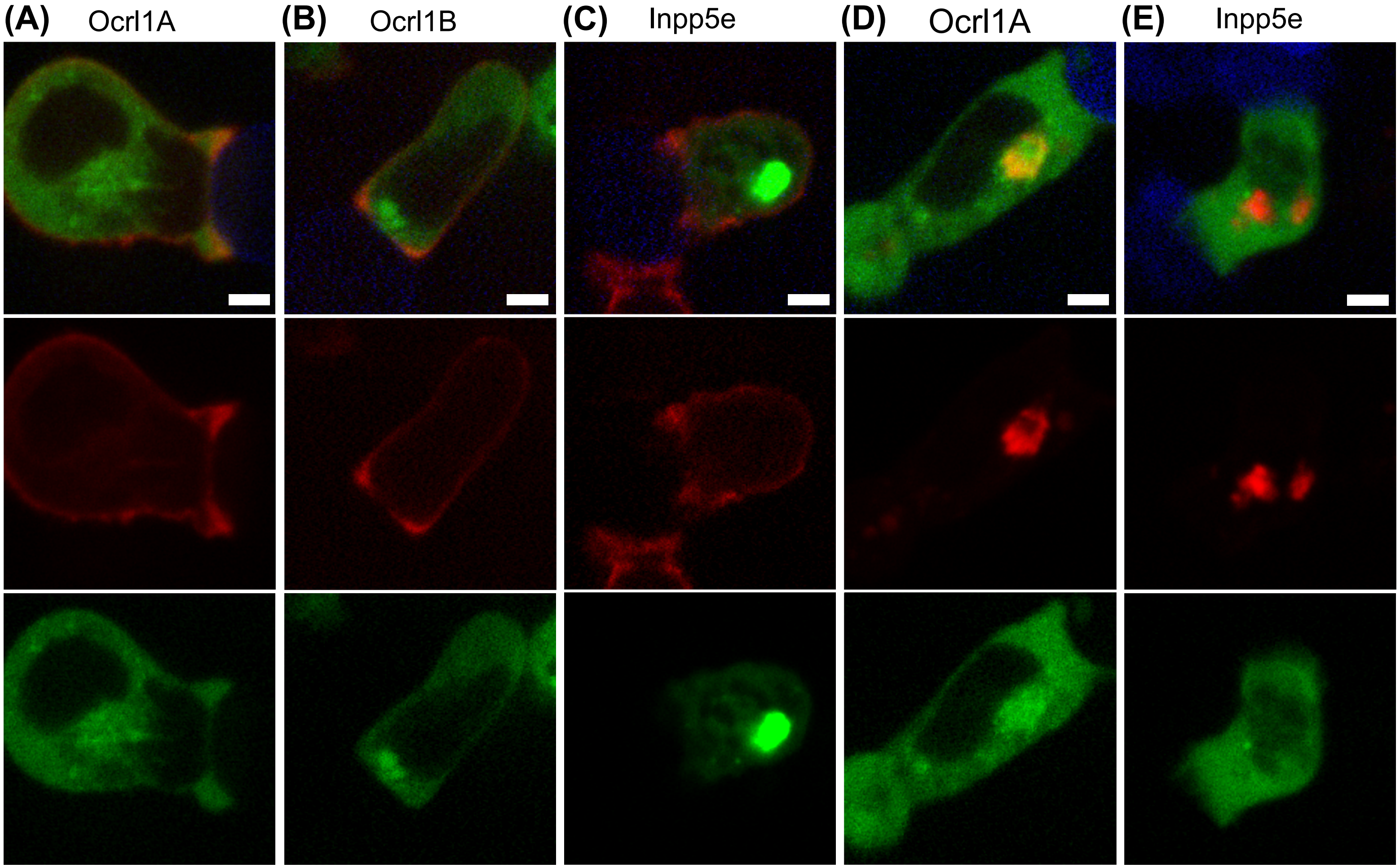

Figure S3. Related to Figure 3 Inpp5e and OCRL1 associate with the Golgi apparatus during synapse formation. Single channel images showing (A) EGFP-Ocrl1A; (B) EGFP-Ocrl1B; (C) EGFP-Inpp5e with Lifeact-mApple (red); (D) EGFP-OCRL1A and (E) EGFP-Inpp5e (green) with Arf1-mTagRFP (red). Scale bars = 3 μm.

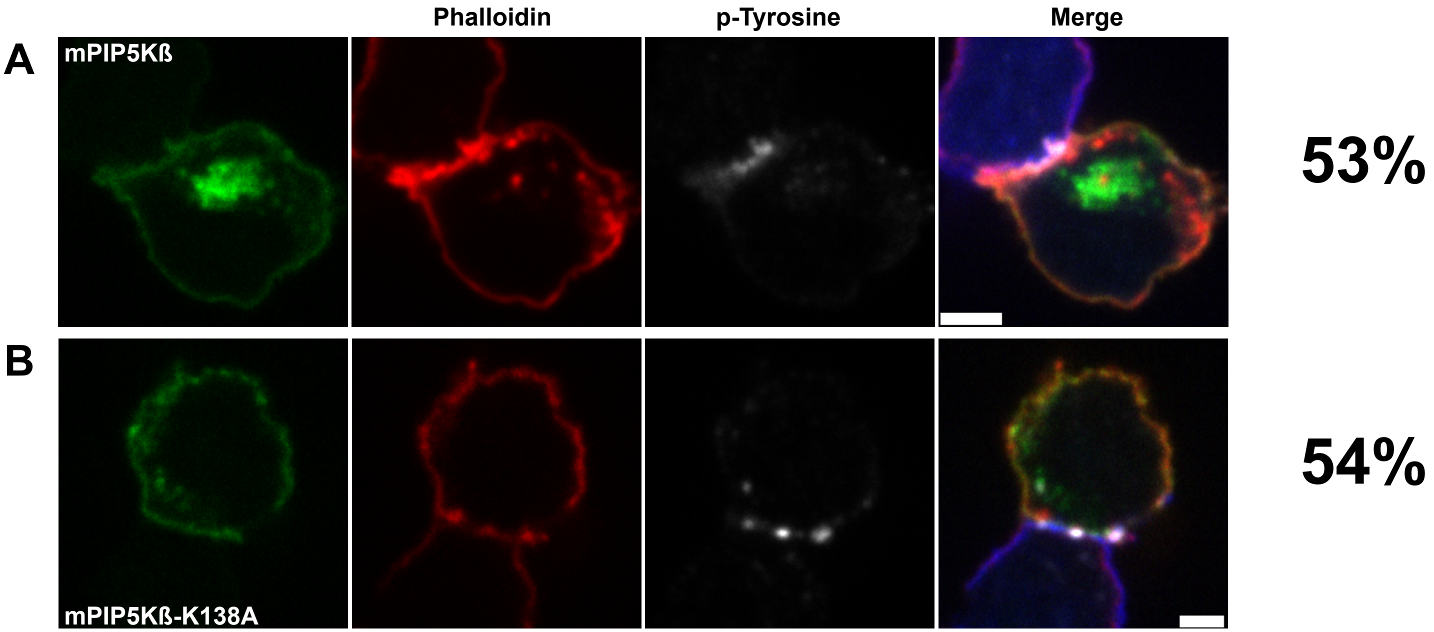

Figure S4 Related to Figure 4 and Figure 5. Frequency of conjugation in OT-I expressing mPIP5K and mPIP5K-K138A. Representative images of CTL expressing (A) mPIP5K (green) or (B) mPIP5K-K138A with EL-4 targets (blue) stained with p-Tyr (white) and Alexa fluor 555 conjugated-phalloidin (red), showing % conjugate frequency (n= 717 mPIP5K; n= 700 mPIP5K-K138A; 3 independent experiments).

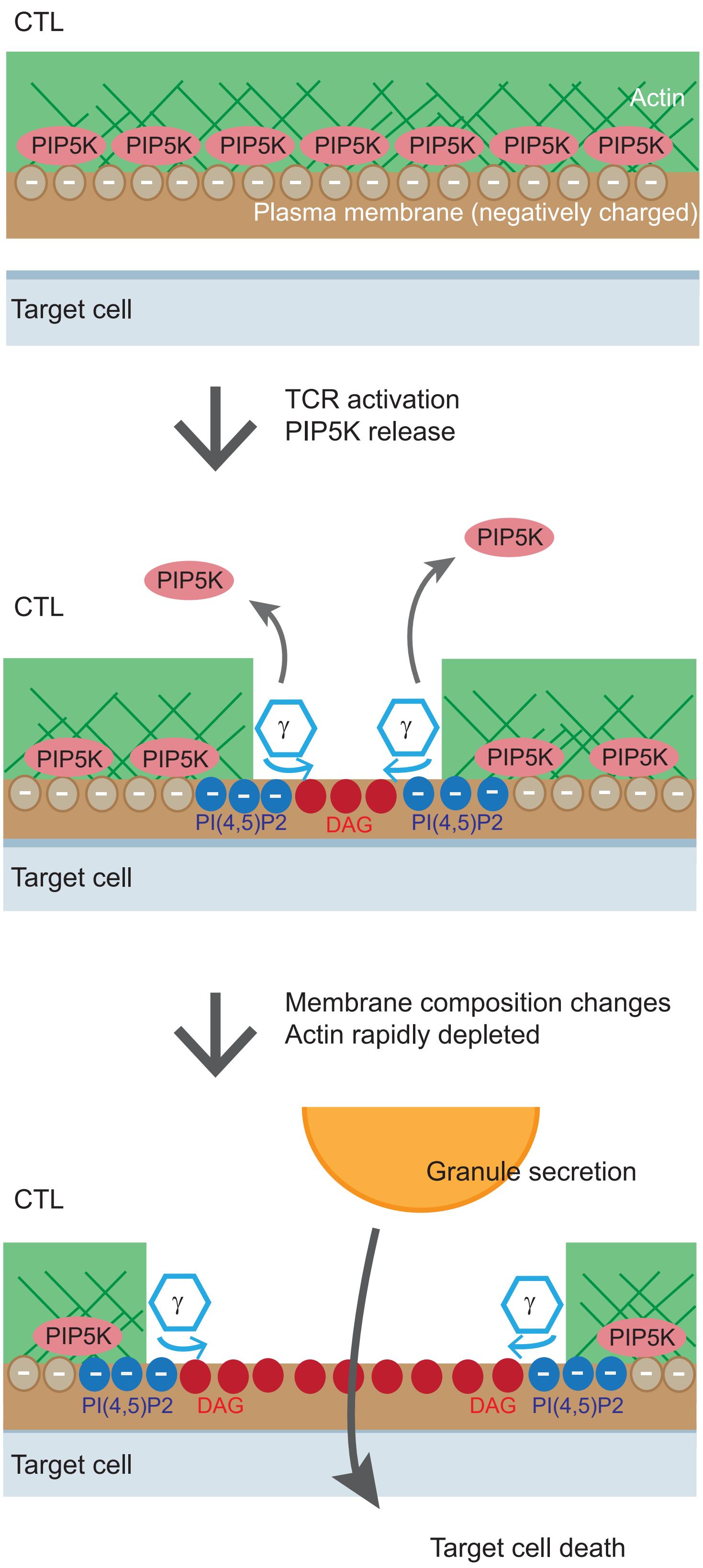

Figure S5 related to Figure 7. Model of PIP5K as a membrane sensor for TCR signalling. PIP5Ks generate PI(4,5)P2 and help maintain negative charge in the CTL membrane (brown). Upon TCR signaling PLCγ1 cleaves PI(4,5)P2 (blue) to generate DAG (red). The reduction in negative charge releases electrostatically-bound PIP5K from the site of signaling with PI(4,5)P2 generation limited to the periphery. Loss of PI(4,5)P2 from the center of the synapse results in the depletion of cortical actin (green), allowing sercretion of granules (orange), destroying the target cell (grey blue).

Supplementary Table 1. Related to figures 1, 2, 3, 6 and & 7. Times to initial increases, depletion and recoveries of bioprobes at the synapse

	Initial increase[1]	Initial depletion[2]	F-actin depletion[2]	Probe recovery[3]	F-actin recovery[3]
PLCγ1-EGFP (n=13)	72 ± 12s<br>Clusters: 74 ± 12s<br>Patch: 161 ± 25s		118 ± 19s	776 ± 95s<br>Patch loss: 708 ± 108s	541 ± 65s
EGFP-PKCγ-C1 (DAG) (n=17)	58 ± 11s		104 ± 15s	358 ± 69s	417 ± 56s
EGFP-Tubby (PI(4,5)P2) (n=19)		116 ± 42s	119 ± 22s	466 ± 41s	466 ± 42s
EGFP-Kras+8 (charge) (n= 15)		116 ± 36s	134 ± 33s	458 ± 80s	516 ± 78s
PIP5KI-EGFP (n=22)		99 ± 10s	106 ± 11s	504 ± 42s	510 ± 41s

[1-3] defined relative to pixel intensities of the probes across the synapse upon initial contact, t=0, with “increase”> pixel intensity at t=0; “depletion” < pixel intensity at t=0; recovery as return to pixel intensity at t=0.
